# In Situ Synthesis of a Stable Fe_3_O_4_@Cellulose Nanocomposite for Efficient Catalytic Degradation of Methylene Blue

**DOI:** 10.3390/nano9020275

**Published:** 2019-02-16

**Authors:** Quan Lu, Yanjuan Zhang, Huayu Hu, Wen Wang, Zuqiang Huang, Dong Chen, Mei Yang, Jing Liang

**Affiliations:** 1 School of Chemistry and Chemical Engineering, Guangxi University, Nanning 530004, China; 15877185376@163.com (Q.L.); yuhuahu@163.com (H.H.); 18260904909@163.com (W.W.); yangmei@gxu.edu.cn (M.Y.); liangl6@gxu.edu.cn (J.L.); 2 State Key Laboratory of Non-Food Biomass and Enzyme Technology, Guangxi Academy of Sciences, Nanning 530007, China; 13878190484@163.com

**Keywords:** cellulose, Fe_3_O_4_ nanoparticles, interaction, catalytic degradation, stable catalyst

## Abstract

To rapidly obtain a stable Fe_3_O_4_@cellulose heterogeneous Fenton catalyst, a novel in situ chemical co-precipitation method was developed. Compared with mechanical activation (MA)-pretreated cellulose (MAC), MA + FeCl_3_ (MAFC)-pretreated cellulose (MAFCC) was more easily dissolved and uniformly distributed in NaOH/urea solvent. MAFCC and MAC solutions were used as precipitators to prepare Fe_3_O_4_@MAFCC and Fe_3_O_4_@MAC nanocomposites, respectively. MAFCC showed stronger interaction and more uniform combination with Fe_3_O_4_ nanoparticles than MAC, implying that MAFC pretreatment enhanced the accessibility, reactivity, and dissolving capacity of cellulose thus, provided reactive sites for the in situ growth of Fe_3_O_4_ nanoparticles on the regenerated cellulose. Additionally, the catalytic performance of Fe_3_O_4_@MAFCC nanocomposite was evaluated by using for catalytic degradation of methylene blue (MB), and Fe_3_O_4_@MAC nanocomposite and Fe_3_O_4_ nanoparticles were used for comparative studies. Fe_3_O_4_@MAFCC nanocomposite exhibited superior catalytic activity for the degradation and mineralization of MB in practical applications. After ten cycles, the structure of Fe_3_O_4_@MAFCC nanocomposite was not significantly changed owing to the strong interaction between MAFCC and Fe_3_O_4_ nanoparticles. This study provides a green pathway to the fabrication of a stable nanocomposite catalyst with high catalytic performance and reusability for the degradation of organic pollutants.

## 1. Introduction

The Fenton reaction, one of the typical advanced oxidation processes (AOPs), has been proven to be one of the most promising alternative wastewater treatment technologies due to its excellent ability to produce strongly reactive hydroxyl radicals, which can attack the organic pollutants and convert the pollutants into small molecules or mineralize them into CO_2_ and H_2_O [1,2]. However, homogeneous Fenton systems have some drawbacks, especially the formation of iron sludge leading to secondary pollution and high cost. On the contrary, heterogeneous Fenton processes have showed great efficiency to overcome these problems [3]. Recently, Fe_3_O_4_ nanocatalyst has attracted more attention because of its unique properties, including excellent magnetism, reusability, and low toxicity [4]. Nevertheless, Fe_3_O_4_ nanoparticles are easy to agglomerate, which will lead to the reduction of their catalytic activity. To preserve the particular performances of Fe_3_O_4_ nanocatalyst, many support materials have been used to immobilize Fe_3_O_4_ nanoparticles to enhance their dispersity, such as activated carbon [5], graphene oxide [6], montmorillonite [7], etc. Additionally, magnetic composites have been widely used as heterogeneous catalysts to treat wastewater. Therefore, it is crucial to select a suitable support for preparing environment-friendly, stable, and renewable supported Fe_3_O_4_ Fenton catalyst for catalytic degradation of organic pollutants in wastewater.

Cellulose is regarded as one of the most abundant organic polymers in nature [8], and has been studied and applied as a precursor of functional materials [9]. It has been reported that cellulose could be used as an excellent support because of its large surface area, good mechanical properties, and almost inexhaustible, biodegradable, and renewable properties [9,10]. Besides, cellulose contains strong inter- and intramolecular hydrogen bonds owing to plenty of hydroxyl groups, which may be an important factor to anchor Fe_3_O_4_ nanoparticles. For instance, Jiao et al. [11] immobilized Fe_3_O_4_ nanoparticles onto cellulose aerogel to prepare Fenton-like catalyst by a hydrothermal method, which displayed a higher degradation rate for Rhodamine B than pure Fe_3_O_4_ nanoparticles. The approach was simply described that cellulose hydrogels were immersed in a mixed iron solution for 24 h in the existence of CH_3_COONa and PEG-4000, and then were heated to 200 °C for 8 h. Qin et al. [12] adopted cellulose nanospheres to support Fe_3_O_4_ nanoparticles through adding two alkaline solutions. Cellulose was treated in NaOH solution for 8 h, and then the composite was precipitated with ammonia aqueous solution. The combination of cellulose nanospheres and Fe_3_O_4_ could remove textile dye rapidly in the existence of H_2_O_2_. Zhu et al. [13] reported the preparation of cellulose/Fe_3_O_4_/activated carbon composite which was applied to adsorption removal of Congo Red. Pure Fe_3_O_4_ was first synthesized, and then Fe_3_O_4_ and activated carbon were added into a cellulose solution to prepare the magnetic adsorbent in the presence of epichlorohydrin. In the aforementioned reports, cellulose acted as encapsulating medium for the magnetic nanoparticles mainly through two processes: monophase cellulose precursor and monophase Fe_3_O_4_ were first prepared separately, and then they were combined to synthesize the composites for dye wastewater treatment. Furthermore, cellulose@Fe_3_O_4_ composite was similarly prepared by multiple processes for the use of other applications [14,15,16]. These sophisticated methods involve high temperatures, long reaction times, use of crosslinker agents, and high cost, which may restrict their structural integrity and practical applications in severe environment. 

Additionally, the high degree of polymerization (DP) of cellulose also hinders its application, because a high DP can prevent the dissolution of cellulose in commonly used solvents. It has been reported that cellulose could rapidly dissolve in a green solvent of 7 wt% NaOH/12 wt% urea aqueous solutions, but the solvent system was also hampered by the high viscosity molecular weight of native cellulose [17,18]. In our previous studies, mechanical activation (MA) and MA + metal salt pretreatments have witnessed the successful destruction of inter- and intramolecular hydrogen bonds and stable crystal structure of native cellulose [19]. Especially, MA + metal salt pretreatment can greatly reduce the DP and crystallinity of cellulose, thus, increase its accessibility and dissolving capacity [8]. Therefore, MA + FeCl_3_ (MAFC) pretreatment was used to destroy the crystal structure and molecular chains of cellulose in this study, which would be beneficial to improve the application of cellulose.

Herein, we present a novel and facile in situ chemical co-precipitation method for the preparation of a stable cellulose supported Fe_3_O_4_ nanoparticles heterogeneous Fenton catalyst without the use of crosslinker agents or intermediate fusion. MAFC-pretreated cellulose (MAFCC) was dissolved in a NaOH/urea solvent system to prepare the cellulose solution, which was used as the alkaline agent and precipitator to prepare the Fe_3_O_4_@MAFCC nanocomposite. In addition, MA-pretreated cellulose (MAC) was also applied to synthesize Fe_3_O_4_@MAC nanocomposite for investigating the effect of pretreatment on the interaction between cellulose and Fe_3_O_4_ nanoparticles. Methylene blue (MB) was selected as a model dye wastewater for the study of catalytic degradation in a heterogeneous Fenton system. The catalytic performance, mineralization capacity, and reusability of Fe_3_O_4_@MAFCC, Fe_3_O_4_@MAC, and pure Fe_3_O_4_ were comprehensively investigated.

## 2. Materials and Methods

### 2.1. Materials 

Bagasse pulp, provided by a local sugar factory (Nanning, China), was used as native cellulose material with a DP of 1010. Anhydrous FeCl_3_, FeCl_3_·6H_2_O, FeCl_2_·4H_2_O, NaOH, H_2_O_2_ (30%), ethanol, H_2_SO_4_ (98%), and tert-butanol were purchased from Guangdong chemical reagents Co. Ltd. (Guangzhou, China). Glucose anhydrous and urea were purchased from Sinopharm Chemical Reagent Co. Ltd. (Shanghai, China). MB was provided by Guangfu Fine Chemical Institute (Tianjin, China). All chemical reagents were of analytical grade and used without further purification. Deionized water was used throughout the experiments.

### 2.2. Pretreatment of Cellulose 

The pretreatment of cellulose was performed according to our previous studies [8,20]. MA + FeCl_3_ (MAFC) and MA were applied to pretreat cellulose, respectively. 500 mL of milling balls (5 mm diameter) was firstly put into a jacketed stainless-steel canister (1200 mL), and then 20 g of dry cellulose and 0.5 g of anhydrous FeCl_3_ were added into the canister. The mixture was stirred for 2 h at a speed of 300 rpm and a temperature of 50 °C by circulating the thermostatic water in the jacket of canister. Finally, MAFCC was obtained by sieving. In addition, MAC was prepared by the same way as MAFCC, without the addition of FeCl_3_.

### 2.3. Preparation of Cellulose@Fe_3_O_4_ Nanocomposite

MAFCC (2.0 g) was added into 7 wt% NaOH/12 wt% urea/81 wt% H_2_O solvent system. The mixed aqueous solution was cooled to −12 °C, and then was immediately thawed and vigorously stirred for 5 min at ambient temperature. The resultant cellulose solution was centrifuged at 9000 rpm for 10 min to remove undissolved cellulose and obtain a transparent cellulose solution.

Fe_3_O_4_@MAFCC nanocomposite was prepared by chemical co-precipitation method where the cellulose solution was used as a precipitator under nitrogen bubbling. FeCl_3_·6H_2_O and FeCl_2_·4H_2_O with Fe^3+^/Fe^2+^ molar ratio of 2:1 (the addition of FeCl_3_·6H_2_O should subtract the amount of FeCl_3_ that added in MAFC pretreatment) and 0.1 g of anhydrous glucose were dispersed in 80 mL water. The aqueous solutions of Fe^3+^ and Fe^2+^ were magnetic stirred for dissolving at 30 °C under nitrogen bubbling. Then the dissolved cellulose solution was added dropwise into the Fe^3+^/Fe^2+^ solution to adjust pH to 12 at 30 °C, followed by constant mechanical stirring to achieve chemical precipitation. After stirring for 30 min, the reaction system was vigorously stirred for 2 h at 80 °C. Consequently, the precipitant was collected by magnetic separation and washed three times using ethanol and distilled water, respectively. Then, the Fe_3_O_4_@MAFCC nanocomposite was freeze dried for 24 h. For comparison, Fe_3_O_4_@MAC nanocomposite was prepared by the same method as Fe_3_O_4_@MAFCC. Pure Fe_3_O_4_ nanoparticle was synthesized by precipitating in 7 wt% NaOH solution under the similar conditions without adding cellulose.

### 2.4. Characterization

The structure of cellulose in NaOH/urea solution was observed by a JEM-2100 transimission electron microscope (TEM, JOELF, Tokyo, Japan). The diluted cellulose solution was suspended on a porous carbon film and dried at ambient temperature, and then the characterization was operated at a voltage of 200 kV. Crystal structure of the samples was measured by a D/MAX2500 V X-ray diffraction (XRD, Rigaku, Tokyo, Japan) using Cu-Kα radiation (*λ* = 0.154 nm) at 40 kV and 30 mA with 2*θ* range from 5° to 80°. The *d* values of the mean diameter of (311) for Fe_3_O_4_ nanoparticles were calculated using the Scherrer equation [21]:

*d* = *kλ*/(*βcosθ*) (1)

where *d* is crystallite size, *k* is a constant applied as 0.89, *λ* is the X-ray wavelength, *β* is the full width at half maximum, and *θ* is the Bragg angle. 

Fourier transform infrared spectroscopy (FTIR) spectra were recorded by a FTIR-8400S Spectrometer (SHIMADZU, Kyoto, Japan) in the range of 400 to 4000 cm^−1^. The surface chemical blinding energies between Fe_3_O_4_ and cellulose were characterized by X-ray photoelectron spectroscopy (XPS, Thermo Fisher Scientific, Waltham, MA, USA). Field emission scanning electron microscopy (FESEM, SUPRA 55 Sapphire, Carl Zeiss, Oberkochen, Germany) was used to analyze the surface morphologies of the samples. A thin layer of gold was coated on the samples to improve the conductivity. Magnetic properties were measured by a Series 7400 model 7404 vibrating sample magnetometer (VSM, LakeShore, Beijing, China), and the hysteretic loop was obtained under an applied magnetic field between −20,000 and 20,000 Oe at 300 K.

### 2.5. Degradation Experiments

MB was used to assess the catalytic properties of Fe_3_O_4_@MAFCC, Fe_3_O_4_@MAC, and Fe_3_O_4_ catalysts in a heterogeneous Fenton reaction as it was one of the most difficult dyes to treat. Typically, 50 mL of MB solution (50 mg L^–1^) was initially adjusted to pH = 2.5 with 0.1 M H_2_SO_4_. Then, the heterogeneous Fenton experiment was performed by adding 0.03 g of catalyst (0.6 g L^–1^) and 0.3 mL H_2_O_2_ (6 mL L^–1^) into 50 mL of MB solution in a thermostat shaker, with a shaking speed of 120 rpm at 50 °C. At different time intervals, the supernatant was drawn and separated rapidly by a magnet. The concentration of remnant MB in supernatant was analyzed by measuring the absorbance of MB at 664 nm on a TU–1901 UV–vis spectrophotometer (Beijing purkinje, Beijing, China). The leaching of Fe from the catalysts was measured according to the 1,10-phenanthroline method [22]. Total organic carbon (TOC) was used to evaluate the degree of mineralization for the heterogeneous Fenton system. Tert-butanol was employed as hydroxyl radical scavenger to determine the generation of •OH species, which played an important role in this catalytic degradation. 

### 2.6. Recyclability Experiments

The wet catalysts were collected by a magnet and washed with deionized water before reused in the next degradation experiment. The degradation procedures were the same as the first degradation experiment during the process of recycling. After ten cycles, the used catalysts were collected and washed with deionized water, and then were freeze-dried for 24 h. The structure and morphology of the used catalysts were determined by XRD and FESEM analyses and compared with those of the fresh catalysts. 

## 3. Results and Discussion

### 3.1. Structure of the Cellulose Solutions 

**Figure 1:**
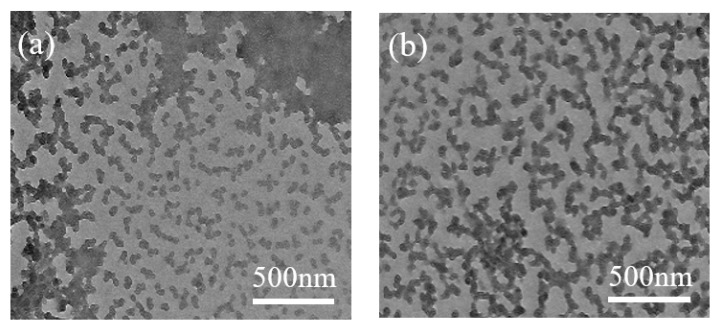
TEM images of (**a**) MAC and (**b**) MAFCC dissolved in NaOH/urea solution.

Previous studies by our group had proved that metal ions could combine with oxygen atoms of hydroxyl groups on the surface of cellulose induced by ball milling to destroy the inter- and intramolecular hydrogen bonds of cellulose, which greatly reduced the DP and crystallinity of cellulose and, thus, improved the dissolution of cellulose [8,23]. As shown in Figure 1a,b, MAC and MAFCC dissolved in NaOH/urea solution display a wormlike pattern, which could be ascribed to that the hydrogen bonds between cellulose and NaOH hydrates were surrounded by urea [24]. Significantly, MAFCC was more uniformly distributed in the solvent than MAC, indicating that MAFC pretreatment enhanced the accessibility of cellulose, which caused a better dispersion in the solvent. As a result, it could be deduced that the uniform distribution of FeCl_3_ on the surface of cellulose by MAFC pretreatment could act as active sites, which was beneficial to prevent the self-aggregation of cellulose chain and enhance the accessibility and reactivity of cellulose. Moreover, the uniform dispersion and combination of the Fe^3+^ ions with the hydroxyl groups of MAFCC could act as anchored sites for in situ growth of Fe_3_O_4_, which could result in the enhanced interaction between cellulose and Fe_3_O_4_. 

### 3.2. Analysis of the Interaction between Cellulose and Fe_3_O_4_

#### 3.2.1. XRD Analysis

**Figure 2:**
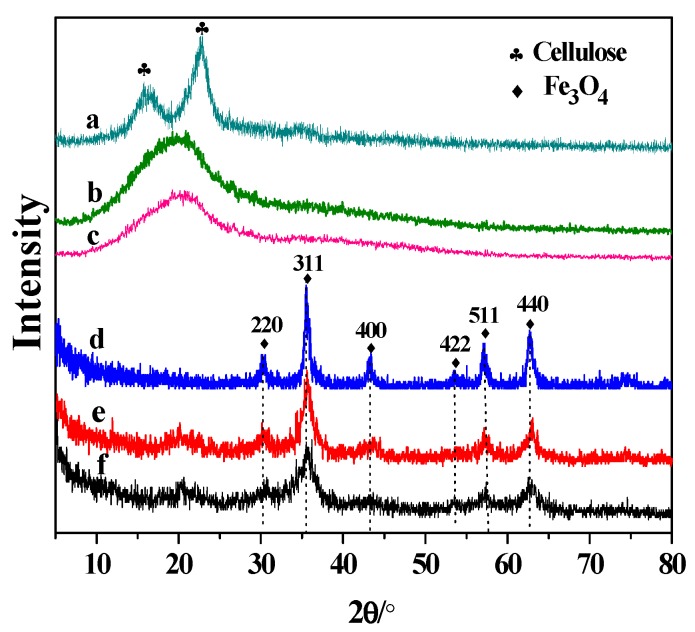
XRD patterns of (a) native cellulose, (b) MAC, (c) MAFCC, (d) Fe_3_O_4_, (e) Fe_3_O_4_@MAC, and (f) Fe_3_O_4_@MAFCC.

XRD analysis was used to investigate the crystal structure of the samples, and the XRD patterns are presented in Figure 2. Figure 2a shows the XRD patterns of native cellulose, which the two strong peaks at 16.2° and 22.8° were assigned to (110) and (200) planes of crystalline cellulose I [23]. Clearly, two distinctive peaks of native cellulose were replaced by a broad band after MA and MAFC pretreatments, and the decrease in the diffraction intensity of MAFCC was more significant compared with that of MAC (Figure 2b,c), confirming that MAFC pretreatment could more remarkably destroy the crystal structure of cellulose, thus, enhance the accessibility and dissolving capacity of cellulose [8]. Additionally, the crystalline structure of the cellulose in the nanocomposites was not obvious (Figure 2e,f), implying that the interaction between Fe_3_O_4_ and cellulose disrupt the crystal structure of cellulose. Moreover, Fe_3_O_4_, Fe_3_O_4_@MAC, and Fe_3_O_4_@MAFCC (Figure 2d–f) exhibit similar diffraction peaks at 30.2°, 35.6°, 43.3°, 53.7°, 57.2°, and 62.8°, which accorded with the (220), (311), (400), (422), (511), and (440) crystal planes with a cubic structure (JCPDS card No. 19-0629) [11,25], suggesting that the anchored Fe_3_O_4_ nanoparticles on the cellulose retained their cubic spinel crystal phase properties. The *d* values of the mean diameter of (311) on Fe_3_O_4_@MAFCC, Fe_3_O_4_@MAC, and Fe_3_O_4_ were 4.16, 7.21, and 15.28 nm, respectively, indicating that the crystallite size of the Fe_3_O_4_ in the nanocomposites was smaller than that of pure Fe_3_O_4_ nanoparticles. The intensity of the diffraction peaks of the nanocomposites was relatively low, resulting from a decrease in the crystalline phase of the Fe_3_O_4_ nanoparticles. Furthermore, the crystallite size and crystalline phase of the Fe_3_O_4_ nanoparticles in the Fe_3_O_4_@MAFCC nanocomposite were weaker than those in the Fe_3_O_4_@MAC nanocomposite, which may attribute to that MAFCC exhibited stronger interaction with Fe_3_O_4_ owing to higher accessibility and reactivity of the cellulose pretreated by MAFC.

#### 3.2.2. FTIR Analysis

**Figure 3:**
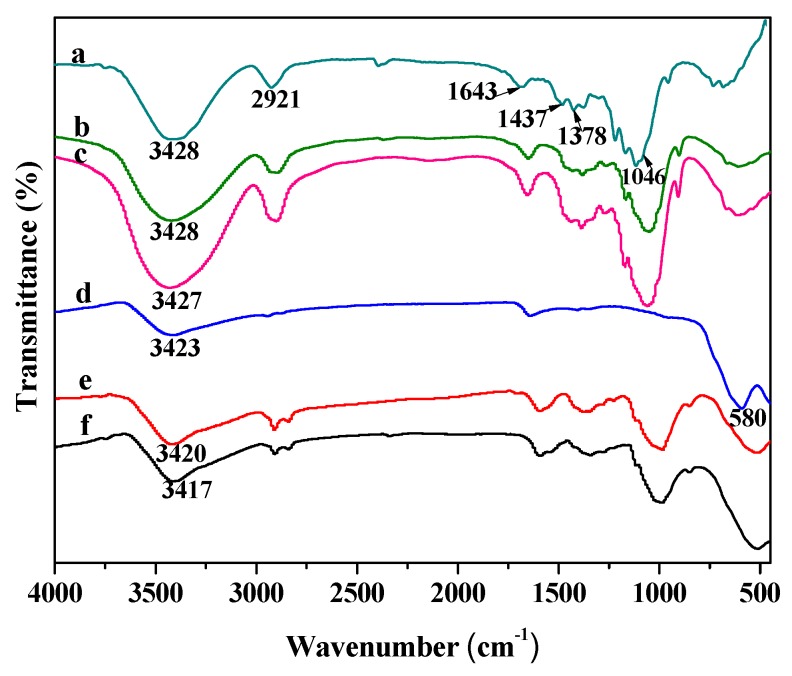
FTIR spectra of (a) native cellulose, (b) MAC, (c) MAFCC, (d) Fe_3_O_4_, (e) Fe_3_O_4_@MAC, and (f) Fe_3_O_4_@MAFCC.

FTIR analysis can reveal some evidence to further confirm the interaction between cellulose and Fe_3_O_4_ nanoparticles. As illustrated in Figure 3, the FTIR spectra of native cellulose, MAC, and MAFCC show the characteristic peaks of cellulose at 3428, 2921, 1437, 1378, and 1046 cm^‒1^, corresponding to the O‒H stretching vibration, C‒H stretching vibration, C‒H banding vibration, C‒H deformation vibration, and C‒O stretching vibration, respectively [12,26]. These characteristic peaks of cellulose were also observed on the spectra of Fe_3_O_4_@MAC and Fe_3_O_4_@MAFCC nanocomposites (Figure 3e,f). The characteristic bands at around 1643 cm^‒1^ ascribed to absorbed water are presented in all samples [27]. In the spectrum of Fe_3_O_4_ (Figure 3d), a peak at 580 cm^‒1^ was the essential characteristic of Fe_3_O_4_ [25], and the peak at 3423 cm^‒1^ was belong to the O‒H bond of water. It was noted that the characteristic peak of Fe_3_O_4_ also displayed on the spectra of the nanocomposites (Figure 3e,f). In particular, the broad peak of the O‒H bond in the nanocomposites shifted to a lower wavenumber compared with that of cellulose, implying the presence of interaction between cellulose and Fe_3_O_4_ through hydrogen bonds [28]. The shift in the spectrum of Fe_3_O_4_@MAFCC was more than that of Fe_3_O_4_@MAC, indicating a stronger interaction appeared in Fe_3_O_4_@MAFCC. It is probably related to that MAFC pretreatment could improve the distribution of Fe^3+^ on the cellulose and the dispersion and accessibility of cellulose in the solvent, leading to a stronger interaction between cellulose and Fe_3_O_4_. These results demonstrate that Fe_3_O_4_ nanoparticles have been immobilized on the cellulose through in situ chemical co-precipitation method, and the MAFC pretreatment is important for enhancing the properties of the Fe_3_O_4_@cellulose nanocomposite.

#### 3.2.3. Surface Element Composition Analysis

**Figure 4:**
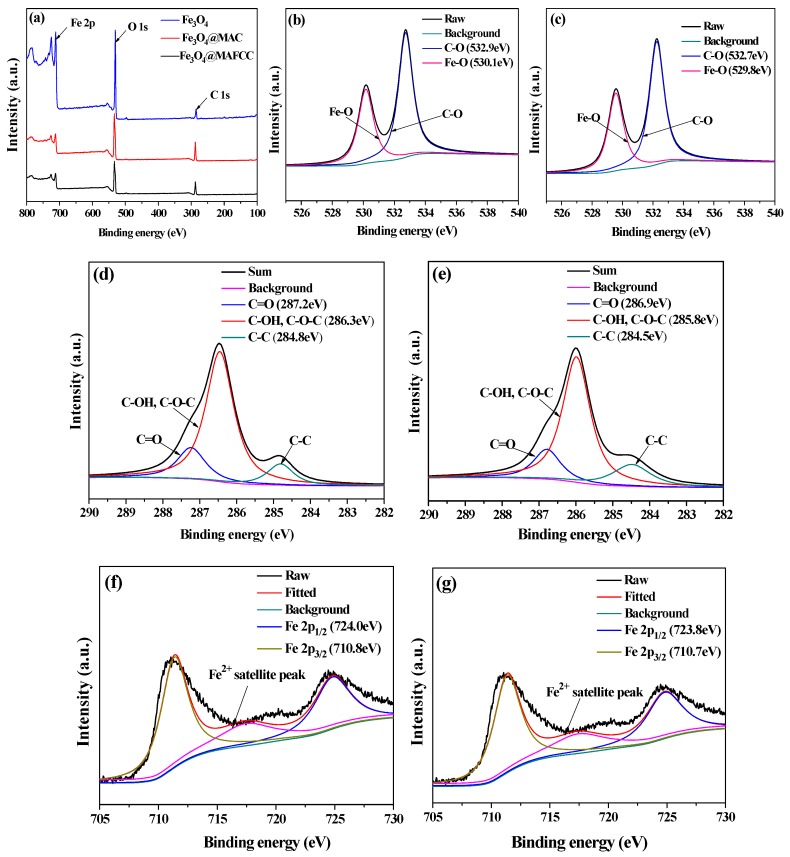
XPS spectra of different samples: (**a**) full-survey of all samples; (**b**,**c**) curve fitting of O 1s spectra of Fe_3_O_4_@MAC and Fe_3_O_4_@MAFCC, respectively; (**d**,**e**) curve fitting of C 1s spectra of Fe_3_O_4_@MAC and Fe_3_O_4_@MAFCC, respectively; (**f**,**g**) curve fitting of Fe 2p spectra of Fe_3_O_4_@MAC and Fe_3_O_4_@MAFCC, respectively.

XPS analysis was performed to investigate the element composition and the surface chemical bonding of the samples. Figure 4a shows the main surface species of all the samples were C, O, and Fe, and no other elements were investigated. Figure 4b,c display the high-resolution O 1s XPS spectra. The binding energies at around 530.1 and 532.9 eV were attributed to Fe‒O bonds with area peak of 38.7% and C‒O bonds with area peak of 61.3% for Fe_3_O_4_@MAC nanocomposite [29,30]. The O 1s spectrum of Fe_3_O_4_@MAFCC was also deconvoluted to two peaks, which were Fe‒O bonds (39.8%) and C‒O bonds (60.2%). These results could indicate that the Fe_3_O_4_ nanoparticles were supported on cellulose. Moreover, the C 1s core-level peak can be fitted into three peaks in Figure 4d. The peaks at 287.2, 286.3, and 284.5 eV for Fe_3_O_4_@MAC were ascribed to C=O, C‒OH or C‒O‒C, and C‒C [11,31], respectively. However, these peaks in Figure 4e were shifted to lower binding energies in the spectrum of Fe_3_O_4_@MAFCC, which may be related to a stronger interaction between MAFCC and Fe_3_O_4_ according to XRD and FTIR analyses. Particularly, these groups would provide many reactive sites for the bonding between Fe_3_O_4_ and cellulose, and the combination mainly through hydroxyl groups on cellulose because the characteristic peak (286.3 eV) was the strongest peaks (Figure 4d). Furthermore, as shown in Figure 4f, the high resolution Fe 2p (10.6%) spectrum of Fe_3_O_4_@MAC contains two peaks at around 710.8 and 724.0 eV, corresponding to Fe 2p_3/2_ and Fe 2p_1/2_ belonging to Fe^3+^ and Fe^2+^ species [32,33], respectively. However, the weak peak of 716.5 eV represented the satellite peak of Fe^2+^ species [34]. The area peaks of Fe^3+^ and Fe^2+^ species were calculated to be 58.6% and 41.4%, respectively. For Fe_3_O_4_@MAFCC (Figure 4g), the Fe 2p (11.8%) spectrum also shows three peaks, which were attributed to Fe^3+^ (54.3%) and Fe^2+^ (45.7%) species. It was found that the atomic concentrations of Fe 2p and area peak of Fe^2+^ for Fe_3_O_4_@MAFCC were more than those of Fe_3_O_4_@MAC, which could lay a foundation for a heterogeneous Fenton reaction. In addition, this analysis could further confirm that the oxide in the nanocomposites was Fe_3_O_4_, which was in good agreement with the reported studies [6,31].

#### 3.2.4. Magnetic Behaviors

**Figure 5:**
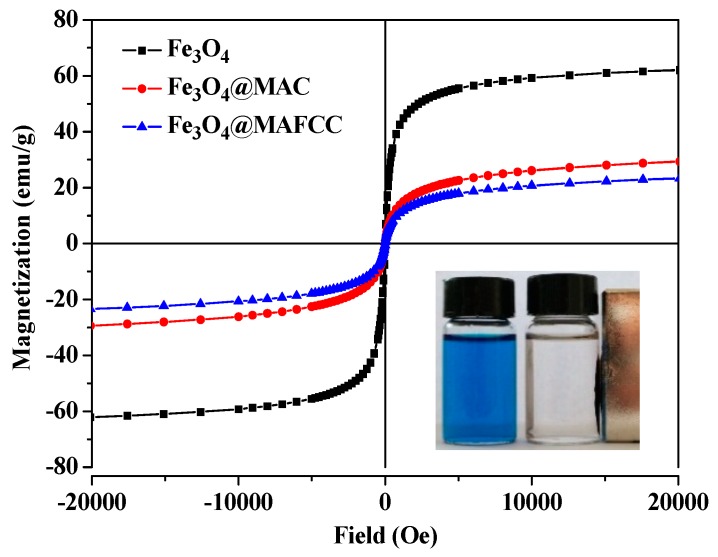
Hysteresis loops of Fe_3_O_4_, Fe_3_O_4_@MAC, and Fe_3_O_4_@MAFCC (inset picture shows the separation of Fe_3_O_4_@MAFCC from the solution by a magnet).

The magnetic properties of different samples were obtained by a vibrating sample magnetometer (VSM) at room temperature. As presented in Figure 5, pure Fe_3_O_4_ nanoparticles performed a higher saturation magnetization (M_S_ = 60.2 emu g^‒1^), while the M_S_ values for Fe_3_O_4_@MAC and Fe_3_O_4_@MAFCC nanocomposites were 29.4 and 23.3 emu g^‒1^, respectively. These mainly due to the fact that cellulose is non-magnetic and thus, reduced the Ms of the nanocomposites, which agreed with other magnetic composites reported by Zhu [13] and Fan [30]. However, the Ms of Fe_3_O_4_@MAFCC was lower than that of Fe_3_O_4_@MAC, which may also be attributed to the strong interaction between MAFCC and Fe_3_O_4_. Despite the reduction of M_S_, the nanocomposites still exhibited superparamagnetic properties, which could be quickly separated from the solution using an external magnetic field, as shown in the inset picture in Figure 5. The magnetic responsivity would be advantageous for their reuse in the treatment of dye wastewater.

#### 3.2.5. Surface Morphology Analysis

**Figure 6:**
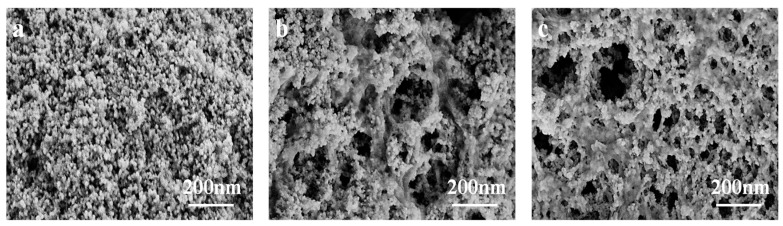
FESEM images of (**a**) Fe_3_O_4_, (**b**) Fe_3_O_4_@MAC, and (**c**) Fe_3_O_4_@MAFCC.

Morphology of the samples was studied by Field emission scanning electron microscopy (FESEM), and the images are shown in Figure 6. It can be seen from Figure 6a that pure Fe_3_O_4_ was the aggregation of sphere-like nanoparticles. In Figure 6b,c, Fe_3_O_4_@MAC and Fe_3_O_4_@MAFCC nanocomposites display porous morphologies, indicating that the presence of cellulose reduced the agglomeration of Fe_3_O_4_ nanoparticles. In particular, Fe_3_O_4_ nanoparticles were evenly distributed on the surface of the regenerated cellulose in the Fe_3_O_4_@MAFCC nanocomposites. This could be ascribed to that the uniform distribution of FeCl_3_ on the surface of cellulose by MAFC pretreatment promoted a good dispersion of cellulose in the solvent and provided reaction sites for the in situ growth of Fe_3_O_4_ nanoparticles, resulting in a uniform combination between cellulose and Fe_3_O_4_ nanoparticles. Meanwhile, the uniform distribution of the Fe_3_O_4_ nanoparticles in cellulose could increase the activities of the nanocomposites, which would help to improve their catalytic properties.

#### 3.2.6. Process of the Combination of Cellulose and Fe_3_O_4_ Nanoparticles

**Figure 7:**
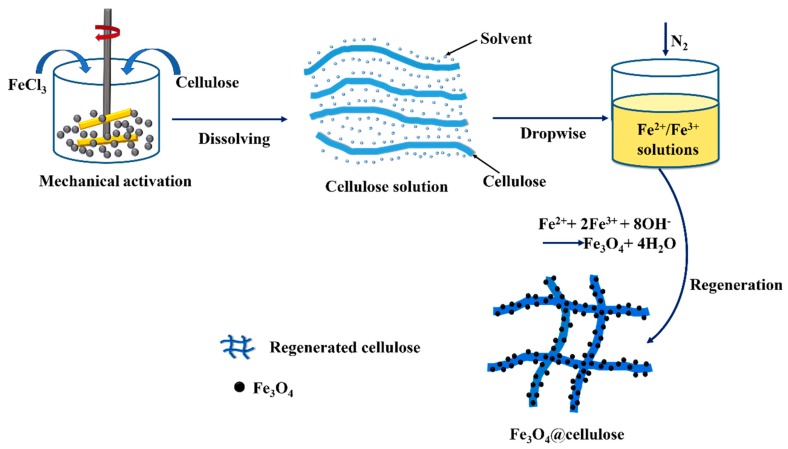
Scheme illustration of the combination of cellulose and Fe_3_O_4_ nanoparticles.

Based on the aforementioned analyses, a reasonable process of the combination of cellulose and Fe_3_O_4_ nanoparticles in the nanocomposites can be illustrated in Figure 7. At first, cellulose was pretreated by MAFC for improving its dissolving capacity. The cellulose displayed a rapid dissolution and a good dispersion in the NaOH/urea solvent, and a wormlike cellulose inclusion complex could be self-assembled with small molecules of the solvent [24]. Afterward, the transparent cellulose solution used as a precipitant was added into the Fe^2+^/Fe^3+^ solution for the formation of Fe_3_O_4_ nanoparticles by chemical co-precipitation method. Simultaneously, the presence of Fe^2+^ and Fe^3+^ ions in the solution and increased temperature could destroy the stability of cellulose solution, resulting in the destruction of the inclusion complex structure of cellulose. Therefore, the cellulose solution could transform into gels by the self-aggregation and entanglement network of cellulose molecules [35,36]. During the process of cellulose regeneration, Fe_3_O_4_ nanoparticles were in situ grown in the regenerated cellulose at the anchored and reactive sites as the uniform distribution of FeCl_3_ on the surface of MAFCC, resulting in an evenly-distributed Fe_3_O_4_ nanoparticles. This novel approach provides a simple method to synthesize eco-friendly nanocomposites and achieves an effective use of resources. Especially, NaOH aqueous solution was not only considered as solvent for the dissolution of cellulose but also used as the precipitator for the preparation of Fe_3_O_4_ nanoparticles in this method.

### 3.3. Catalytic Degradation of MB

**Figure 8:**
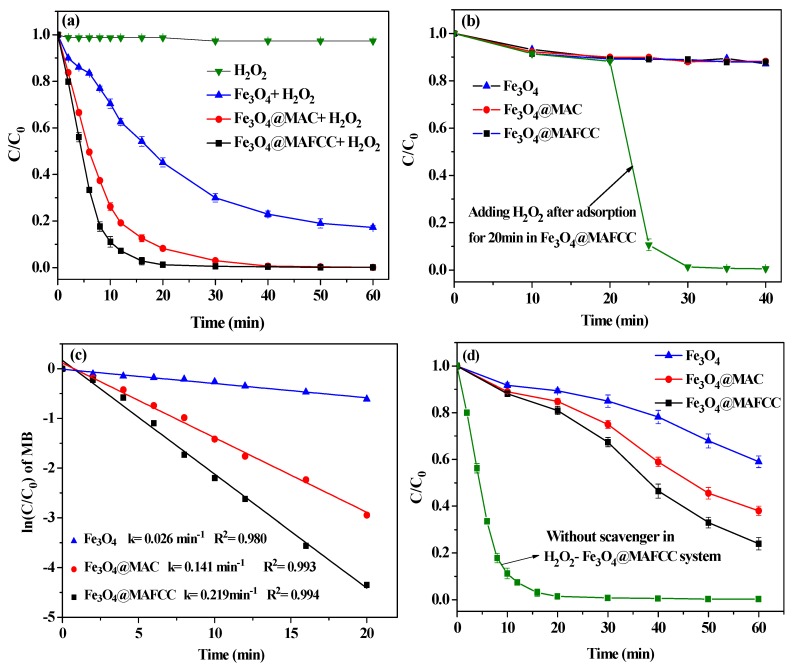
(**a**) Degradation of MB without and with different catalysts in the presence of H_2_O_2_; (**b**) adsorption performance of different catalysts without H_2_O_2_ and catalytic performance of Fe_3_O_4_@MAFCC with adding H_2_O_2_ after adsorption for 20 min; (**c**) pseudo-first-order kinetics fitting results for the degradation of MB with different catalysts; (**d**) degradation of MB with different catalysts in the presence of radical scavenger and H_2_O_2_ and without scavenger in H_2_O_2_-Fe_3_O_4_@MAFCC system. (*C*_0_(MB) = 50 mg L^–1^, *C*(catalysts) = 0.6 g L^–1^, *C*(H_2_O_2_) = 6 mL L^–1^, pH = 2.5).

Heterogeneous Fenton degradation of MB was chosen to evaluate the catalytic activity of the as-prepared samples. As shown in Figure 8a, the degradation of MB by only H_2_O_2_ was almost negligible, indicating that the lack of degradation of the dye discards any visible light induced reactivity [37]. With the addition of catalysts, a significant reduction of the MB concentration was observed. After reacting for 20 min, only 42.7% of MB was degraded in the presence of Fe_3_O_4_ and H_2_O_2_, while the degradation of MB achieved 90.8% and 98.2% when using Fe_3_O_4_@MAC and Fe_3_O_4_@MAFCC as the catalysts, respectively. It was found that the introduction of cellulose obviously enhanced the catalytic activity and a higher degradation was obtained in H_2_O_2_-Fe_3_O_4_@MAFCC heterogeneous Fenton system than in H_2_O_2_-Fe_3_O_4_@MAC heterogeneous Fenton system. On the other hand, Figure 8b shows the adsorption curves of MB on all the samples. When only the catalysts existed in the MB solution, C_t_/C_0_ decreased to 0.88 at 20 min, demonstrating that about 13% of MB was adsorbed by the catalysts. The adsorption capacity of these three catalysts exhibited no obvious difference. However, after adsorbing for 20 min by Fe_3_O_4_@MAFCC catalyst, the concentration of MB sharply decreased as the addition of H_2_O_2_ after reacting for only 5 min, indicating that the catalysis included two processes, the adsorption of MB onto the catalysts and the catalytic degradation of the adsorbed MB. Evidently, catalytic degradation played a crucial role in the removal of MB.

To verify the excellent catalytic action in H_2_O_2_-Fe_3_O_4_@MAFCC heterogeneous Fenton system in comparison with other systems, the pseudo-first-order kinetics was adopted to simulate the degradation rate of MB as follows:

ln(*C*/*C*_0_) = −*kt* (2)

where *C* represents the concentration of MB at time *t*, *C*_0_ is the initial concentration of MB, and *k* is the apparent pseudo first order rate constant. As shown in Figure 8c, a fitting data of ln(*C*/*C*_0_) versus time (0–20 min) is linear for different catalysts, and the correlation coefficient *R*^2^ ≥ 0.98, suggesting that the degradation of MB by these catalysts followed a pseudo first order kinetics model. The *k* values for Fe_3_O_4_, Fe_3_O_4_@MAC, and Fe_3_O_4_@MAFCC catalysts were calculated to be 0.026, 0.141, and 0.219 min^‒1^, respectively. Clearly, Fe_3_O_4_@MAFCC exhibited a maximum rate constant, which could further prove that Fe_3_O_4_@MAFCC showed the best catalytic performance among these catalysts. This phenomenon could be ascribed to the smaller crystallite size of Fe_3_O_4_, higher atomic concentration of Fe 2p and area peak of Fe^2+^, and the uniform distribution of Fe_3_O_4_ in Fe_3_O_4_@MAFCC nanocomposite, which could accelerate the contact between Fe_3_O_4_ and H_2_O_2_ to generate active sites [11]. Therefore, H_2_O_2_-Fe_3_O_4_@MAFCC heterogeneous Fenton system may provide numerous reactive sites to greatly improve the catalytic degradation of MB. 

As reported in many studies, hydroxyl radicals (•OH) are the master active species in the whole heterogeneous Fenton degradation process, which display a much higher oxidation potentials than •O_2_^−^ and •OOH [12,38]. Tert-butanol is a common radical scavenger to determine whether the catalytic degradation involved •OH. As presented in Figure 8d, with the addition of 0.6 mol L^‒1^ tert-butanol, the degradation rate of MB was severely inhibited by comparison with that without tert-butanol in H_2_O_2_-Fe_3_O_4_@MAFCC system, suggesting that the severely quenched production of •OH imposed a significant impact on the reaction process. Additionally, it is evident that the degradation of MB was greatly inhibited in H_2_O_2_-Fe_3_O_4_@MAC and H_2_O_2_-Fe_3_O_4_ systems than in H_2_O_2_-Fe_3_O_4_@MAFCC system, indicating that the H_2_O_2_-Fe_3_O_4_@MAFCC heterogeneous Fenton system could produce more •OH to enhance the degradation rate of MB.

As mentioned above, a process for the degradation of MB was explained in the following reactions [38,39,40]:

≡Fe^III^ + H_2_O_2_ → ≡Fe^III^ H_2_O_2_ (3)

≡Fe^III^ H_2_O_2_ → ≡Fe^II^ + •O_2_H + H^+^ (4)

≡Fe^III^ + •O_2_H → ≡Fe^II^ + O_2_ + H^+^ (5)

≡Fe^II^ + H_2_O_2_ → ≡Fe^III^ + •OH + OH^−^ (6)

**Figure 9:**
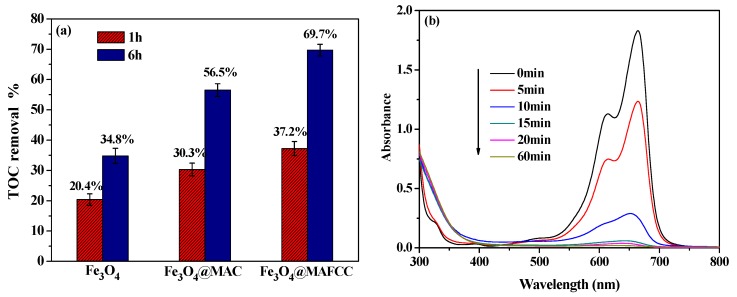
(**a**) TOC removal of the MB solution catalyzed by different catalysts and (**b**) temporal evolution of the UV–vis spectra during the degradation of MB in the H_2_O_2_-Fe_3_O_4_@MAFCC heterogeneous Fenton system. (*C*_0_ (MB) = 50 mg L^–1^, *C*(catalysts) = 0.6 g L^–1^, *C*(H_2_O_2_) = 6 mL L^–1^, pH = 2.5).

•OH + MB →low molecular substances + CO_2_ + H_2_O (7)

the initial stage of the reaction was the formation of a composite of ≡Fe^III^ H_2_O_2_, where ≡Fe^III^ stands for Fe (III) sites on the surface of the catalysts (Equation (3)). Afterward, ≡Fe^III^ species could be reduced to ≡Fe^II^ species (Equations (4) and (5)), and the ≡Fe^II^ species were the main reactive sites to catalyze the activation of H_2_O_2_ for generating •OH radicals (Equation (6)), resulting in effective degradation of MB molecules to low molecular substances and even mineralization of MB to CO_2_ and H_2_O (Equation (7)). In this study, the mineralization of MB in different systems was measured and shown in Figure 9a. During the initial 60 min, the TOC removal efficiencies were 37.2%, 30.3%, and 20.4% with Fe_3_O_4_@MAFCC, Fe_3_O_4_@MAC, and Fe_3_O_4_ as catalysts, respectively. When the reaction proceeded to 6 h, the TOC removal increased significantly. In contrast, Fe_3_O_4_@MAFCC displayed a greater mineralization of MB in the reaction. To analyze the difference in the degradation and mineralization of MB, the temporal evolution of the degradation of MB in the H_2_O_2_-Fe_3_O_4_@MAFCC heterogeneous Fenton system was presented in Figure 9b. The maximum absorption wavelength at 665 nm was ascribed to the functional groups of the chromophore of MB and its dimers, which were attributed to ‒C=S and ‒C=N [41]. Notably, the intensity of the peak was obviously decreased and almost disappeared with the reaction time of only 20 min, indicating that the rapid destruction on the conjugate structure of MB molecules. A high degradation efficiency (98.2%) was obtained at a short time, because the chromophore groups of MB molecules were broken down rapidly, but TOC removal (37.2%) was relatively slower. Therefore, the process of mineralization fell behind the procedure of degradation. Nevertheless, a high TOC removal could be achieved by prolonging reaction time in this study. The extent of the mineralization efficiency of MB was an evidence for the practical applications of the catalysts.

### 3.4. Reusability Analysis

**Figure 10:**
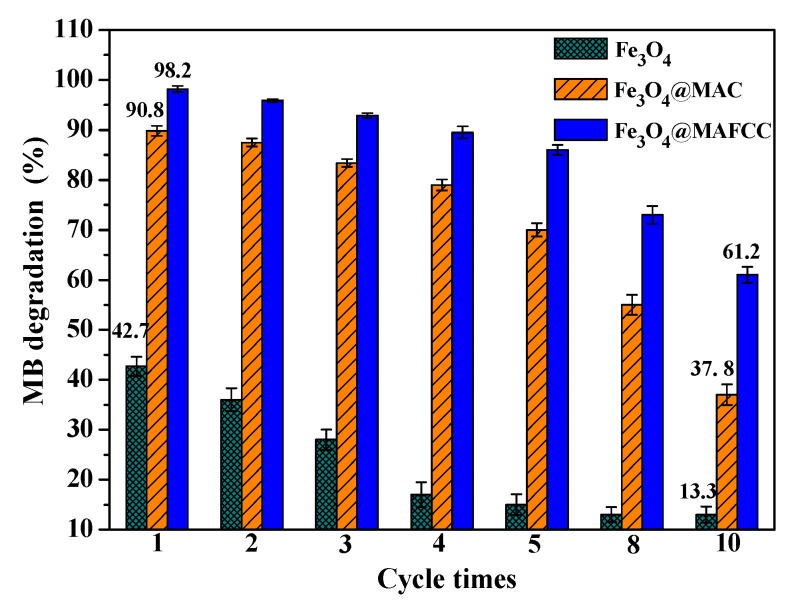
Degradation efficiency of MB catalyzed by Fe_3_O_4_, Fe_3_O_4_@MAC, and Fe_3_O_4_@MAFCC recycled for different times. (*C*_0_ (MB) = 50 mg L^–1^, *C*(catalysts) = 0.6 g L^–1^, *C*(H_2_O_2_) = 6 mL L^–1^, pH = 2.5).

The reusability analysis was carried out to evaluate the potential applications of catalysts. Figure 10 shows the degradation of MB catalyzed by different catalysts for ten cycles. It is noted that the nanocomposites showed better reusability than pure Fe_3_O_4_ nanoparticles for each cycle, demonstrating that the catalysts in the presence of cellulose loaded with MB could be regenerated and reused several times [42]. However, when the number of cycles increased to ten times, the catalytic performance of the nanocomposites appeared different degree of reduction. The degradation efficiency of MB catalyzed by reused Fe_3_O_4_@MAFCC (61.2%) was markedly higher than that by reused Fe_3_O_4_@MAC (37.8%) at the tenth cycle, which could be attributed to the strong interaction between MAFCC and Fe_3_O_4_, leading to a better reusability of the catalyst. In addition, catalyst deactivation may be related to the adsorption of organic intermediates reducing the active catalytic sites and the loss of catalysts during the reaction and rinsing process [12]. Thus, it is important to investigate the causes of catalyst deactivation.

**Figure 11:**
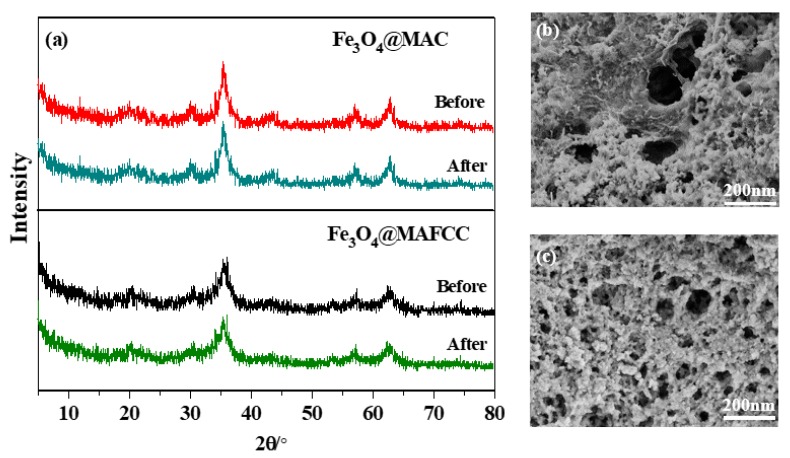
(**a**) XRD patterns of Fe_3_O_4_@MAC and Fe_3_O_4_@MAFCC before and after ten cycles; (**b**) FESEM image of the recycled Fe_3_O_4_@MAC after ten cycles; (**c**) FESEM image of the recycled Fe_3_O_4_@MAFCC after ten cycles.

To clearly analyze the structural properties of the catalysts after catalytic degradation of MB, XRD and FESEM analyses were used to further prove the stability of the catalysts. As can be seen in Figure 11a, the crystal phase of the recycled Fe_3_O_4_@MAFCC and Fe_3_O_4_@MAC catalysts were the same as that of the original ones. However, the morphology of the used Fe_3_O_4_@MAC catalyst was different from that of the fresh one (Figure 11b). The interface between cellulose and Fe_3_O_4_ nanoparticles was easier to be observed from the recycled Fe_3_O_4_@MAC nanocomposite, which may be associated with the shedding of the Fe_3_O_4_ nanoparticles from the surface of cellulose during degradation experiments. This may be one of the reasons for the deactivation of catalyst. Particularly, the FESEM image of the recycled Fe_3_O_4_@MAFCC nanocomposite did not remarkably change after ten cycles (Figure 11c), which was also the porous morphology with uniform combination of the Fe_3_O_4_ nanoparticles on the surface of cellulose. These results show that the stability of Fe_3_O_4_@MAFCC catalyst was higher than that of Fe_3_O_4_@MAC catalyst, which was mainly owing to the strong interaction between MAFCC and Fe_3_O_4_. In addition, the level of iron leaching for Fe_3_O_4_@MAFCC catalyst was calculated to be lower than that of Fe_3_O_4_@MAC catalyst, but the amount was lower than 2 ppm, which conforms to the European standard [43]. Therefore, Fe_3_O_4_@MAFCC nanocomposite could be considered as an efficient and stable catalyst for the degradation and mineralization of the organic pollutants in practical applications.

## 4. Conclusions

In summary, a stable cellulose supported Fe_3_O_4_ nanocomposite was successfully synthesized through a facile in situ chemical co-precipitation method with cellulose solution as a precipitator for the catalytic degradation of MB. Using Fe_3_O_4_@MAC nanocomposite as a comparison, the cellulose pretreated by MAFC showed a good dispersion in the solvent and enhanced accessibility and reactivity, resulting in a uniform combination and strong interaction between MAFCC and Fe_3_O_4_ nanoparticles. The uniform distribution of FeCl_3_ on the surface of cellulose by MAFC pretreatment could provide reactive sites for the in situ growth of Fe_3_O_4_ nanoparticles in the regenerated cellulose. Moreover, Fe_3_O_4_@MAFCC catalyst could produce more •OH to enhance the catalytic degradation and mineralization of MB in the presence of H_2_O_2_, which was mainly attributed to the uniform dispersion of Fe_3_O_4_ nanoparticles on the cellulose. Additionally, the Fe_3_O_4_@MAFCC catalyst exhibited better reusability and stability than Fe_3_O_4_@MAC catalyst even after ten cycles, owing to the stronger interaction between MAFCC and Fe_3_O_4_ nanoparticles. Consequently, this novel approach could make full use of renewable resources to prepare stable nanocomposite catalyst for practical application of organic pollutants.
